# Biomechanical Consequences of Rapid Evolution in the Polar Bear Lineage

**DOI:** 10.1371/journal.pone.0013870

**Published:** 2010-11-05

**Authors:** Graham J. Slater, Borja Figueirido, Leeann Louis, Paul Yang, Blaire Van Valkenburgh

**Affiliations:** 1 Department of Ecology and Evolutionary Biology, University of California Los Angeles, Los Angeles, California, United States of America; 2 Departamento de Ecología y Geología, Área de Paleontología, Facultad de Ciencias, Universidad de Málaga, Campus Universitario de Teatinos, Málaga, Spain; 3 Cornell University Museum of Vertebrates, Ithaca, New York, United States of America; Raymond M. Alf Museum of Paleontology, United States of America

## Abstract

The polar bear is the only living ursid with a fully carnivorous diet. Despite a number of well-documented craniodental adaptations for a diet of seal flesh and blubber, molecular and paleontological data indicate that this morphologically distinct species evolved less than a million years ago from the omnivorous brown bear. To better understand the evolution of this dietary specialization, we used phylogenetic tests to estimate the rate of morphological specialization in polar bears. We then used finite element analysis (FEA) to compare the limits of feeding performance in the polar bear skull to that of the phylogenetically and geographically close brown bear. Results indicate that extremely rapid evolution of semi-aquatic adaptations and dietary specialization in the polar bear lineage produced a cranial morphology that is weaker than that of brown bears and less suited to processing tough omnivorous or herbivorous diets. Our results suggest that continuation of current climate trends could affect polar bears by not only eliminating their primary food source, but also through competition with northward advancing, generalized brown populations for resources that they are ill-equipped to utilize.

## Introduction

**Figure 1:**
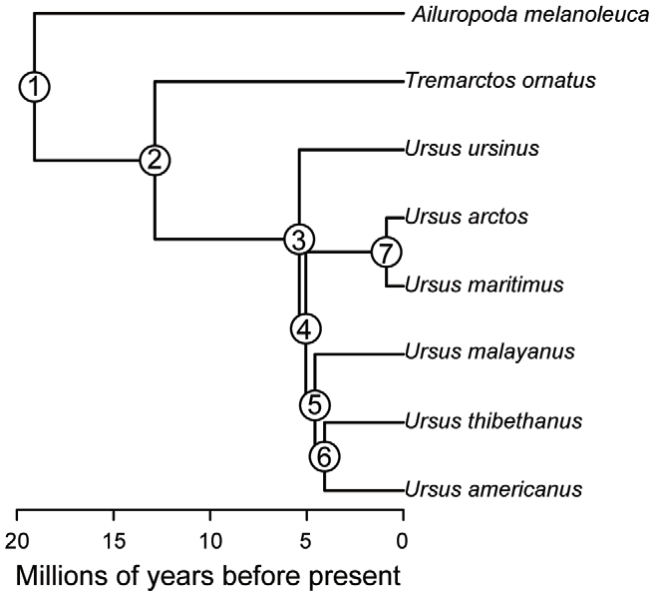
Time-calibrated ursid phylogeny used for assessing rates of cranial shape evolution. Node numbers correspond to those used for evolutionary contrasts (see Table 1).

The polar bear *Ursus maritimus* is unique among living ursids as the only member of the family with an exclusively carnivorous diet. As a result of this specialized diet, the the polar bear has evolved a series of craniodental adaptations that allow it to efficiently process a diet of seal flesh and blubber. For example, polar bears exhibit reduced surface area of the grinding molar teeth, a feature normally pronounced in more omnivorous ursids, and a low, slender skull [1], [2]. Despite possessing such distinctive phenotypic features, molecular and paleontological data unequivocally indicate that the carnivorous polar bear evolved relatively recently, approximately 150–700ka (Fig. 1), from coastal populations of the more generalized and omnivorous brown bear *Ursus arctos* [3]–[6]. In this study, we take a combined evolutionary and biomechanical approach to examine the evolution of adaptations to carnivory in the polar bear cranium. We first use multivariate evolutionary contrasts [7] to test whether the unique cranial morphology of the polar bear resulted from increased rates of cranial shape evolution in the polar bear lineage, relative to other branches of ursid phylogeny. We expect this to be the case if adaptation to the harsh arctic environment and a hypercarnivorous diet posed novel evolutionary challenges for a large ursid. We then use 3D finite element analysis (FEA) to examine the impact of craniodental adaptations to hypercarnivory on various aspects of cranial performance, such as bite force and skull strength, during feeding. FEA is an engineering method used to examine patterns of stress and strain in man-made objects when placed under load and, in recent years, has been adapted to study the evolution of biological form and function [8]–[14]. In FEA, the structure of interest, here the skull, is represented as a finite number of elements, joined at their vertices by nodes. The elements are assigned material properties that specify how they respond when placed under load. Recent developments in FE modeling of biological structures have resulted in methods for more realistic modeling of jaw muscles [15] and appropriate protocols for assessing comparative performance across species [16]. Here, we use FEA to compare feeding performance in the carnivorous polar bear to that of its phylogenetically and geographically closest relative, the omnivorous brown bear.

## Results

Multivariate rates of evolution for cranial shape are given in Table 1, where node numbers refer to nodes in Figure 1. The rate of skull shape evolution in the polar bear lineage was about double the mean rate observed for other parts of ursid phylogeny (mean ursid rate = 0.024, +/−0.007, polar bear rate = 0.059). This difference was significant based on a one-tailed T-test (t*_6_* = 4.92, *p* = 0.0013).

**Table 1:** Mulitvariate evolutionary contrasts for ursid cranial shape.

Contrast	mulitvariate rate
*A. melanoleuca* / node 1	0.025
*T. ornatus* / node 2	0.013
*U.ursinus* / node 3	0.023
*U. arctos* / node 4	0.032
*U.malayanus* / node 5	0.030
*U. thibethanus / U.americanus*	0.018
Ursid mean (sd)	0.024 (0.007)
*U.maritimus / node 7*	0.059

Node numbers refer to nodes in Fig. 1.

**Figure 2:**
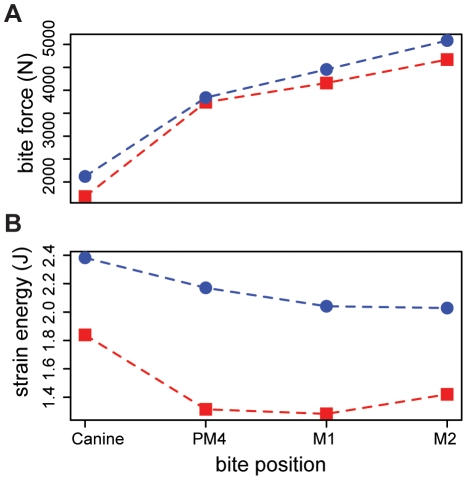
Performance metrics assessed for four different bite positions in the polar bear (blue symbols) and brown bear (red symbols) FE models. The *X* axis corresponds to bite point, with anterior bites towards the left and posterior bites to the right. Panel A shows bite forces, panel B shows cranial strain energy. Note that bite forces are similar in both species for all bites, while strain energies are uniformly lower in the brown bear.

Surface area to volume ratios for the finite element models of polar and brown bear skulls were similar, indicating that similar amounts of bone are used in the skulls of both species (SA/V: polar bear = 0.61, brown bear = 0.59). This finding suggests that differences in stress magnitudes between the polar bear and scaled brown bear skull models can be interpreted in light of differences in external shapes of the skulls. Bite forces measured from the two scaled finite element models were also comparable for all simulated bites, although the polar bear's bite was slightly stronger in each case (Table 2, Fig. 2a). These results suggest that the potential leverage of the jaw muscle systems is also similar for the two species.

**Table 2:** Bite forces and strain energy density (SED) values for the two models under four simulated bites.

		Canine	PM4	M1	M2
*polar bear*					
	Bite Force right	1939.96	3798.69	4481.55	5041.65
	Bite Force left	2302.09	3882.04	4426.24	5127.36
	mean Bite force	2121.02	3840.36	4453.90	5084.50
	SED right	2.38	2.36	1.91	2.03
	SED left	-	1.98	2.17	2.03
	mean SED	2.38	2.17	2.04	2.03
*brown bear*	Bite Force right	1731.92	3832.53	4197.82	4570.43
	Bite Force left	1630.84	3644.36	4119.48	4768.38
	mean Bite force	1681.38	3738.45	4158.65	4669.41
	SED right	1.86	1.31	1.25	1.52
	SED left	-	1.35	1.34	1.35
	mean SED	1.86	1.33	1.30	1.44

Values are given for FE analyses conducted with bite points on the right and left sides, as well as means over both sides.

**Figure 3:**
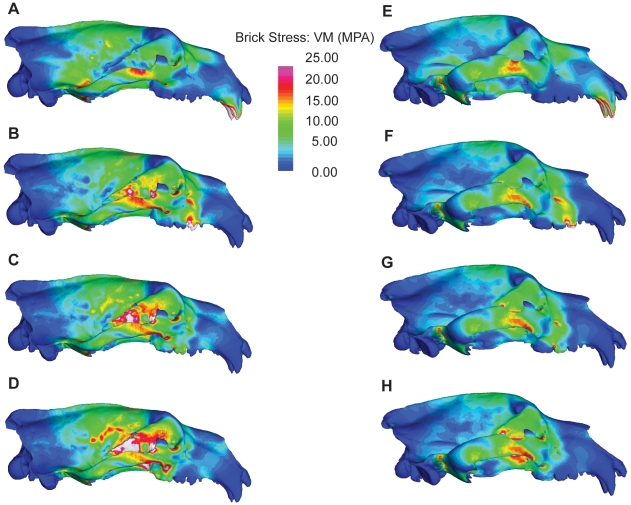
FE models showing von Mises stresses in the polar bear (left) and brown bear (right) skulls during bilateral canine biting (A,E), and unilateral PM4 (B,F), M1 (C,G), and M2 (D,H) biting.

Stress distributions and magnitudes differed between the two models for all bites. For each biting scenario, the polar bear skull exhibited more widely varying stresses (Fig. 3) and higher peak stresses (Table 3) than for the brown bear. Differences between the two species were most marked for bites made with the molars, where peak stresses in the polar bear were up to 408% those of the brown bear (Table 3). Similarly strain energy values were higher in the polar bear cranium than for the brown bear for all bites (Table 2; Fig. 2b), indicating that the polar bear skull undergoes more deformation in producing similar bite forces. Again, differences between the polar and brown bear crania were most pronounced for bites made with the post-canine dentition, the main site for processing of ingested food. Our model results are unvalidated by *in vivo* data and should be treated as estimates only. However, based on our findings, it appears that although the two species are similar in cranial size and have similar muscle leverage potential, the polar bear's skull is a weaker, less work-efficient structure, and does not appear well suited to dealing with large masticatory loads.

**Table 3:** Peak Von mises stress for homologous cranial regions in the polar and brown bear models for the four simulated bites.

bite position	skull region	polar bear	brown bear
bilateral canine	rt zygoma	29.13 (7.95)	26.46 (6.73)
	lt zygoma	34.77(8.45)	27.08 (6.60)
	palate	24.49 (5.66)	10.50 (2.94)
	snout	13.49 (4.51)	10.66 (4.59)
	frontal	31.53 (6.32)	11.66 (4.84)
	rt orbit	27.72 (6.85)	15.75 (5.13)
	lt orbit	19.88 (7.32)	21.40 (3.80)
PM4	rt zygoma	29.92 (9.30)	21.02 (6.93)
	lt zygoma	25.27 (5.65)	19.17 (4.54)
	palate	21.20 (5.12)	9.94 (2.74)
	snout	20.39 (2.76)	14.41 (2.58)
	frontal	24.39 (4.65)	10.49 (4.03)
	rt orbit	62.31 (15.37)	20.23 (8.64)
	lt orbit	14.07 (3.75)	11.63 (2.02)
M1	rt zygoma	38.15 (9.79)	22.84 (7.04)
	lt zygoma	25.23 (6.08)	19.16 (4.61)
	palate	52.84 (6.76)	16.10 (3.24)
	snout	13.81 (2.66)	13.94 (2.14)
	frontal	22.9 (4.03)	10.47 (3.85)
	rt orbit	114.18 (23.22)	27.93 (11.03)
	lt orbit	21.84 (3.55)	9.87 (1.84)
M2	rt zygoma	38.81 (9.92)	27.26 (7.66)
	lt zygoma	24.75 (4.63)	18.80 (4.58)
	palate	52.93 (6.51)	17.93 (3.37)
	snout	13.36 (2.63)	10.99 (1.77)
	frontal	22.92 (3.64)	11.09 (3.83)
	rt orbit	114.63 (23.16)	45.72 (15.62)
	lt orbit	22.09 (3.63)	11.58 (1.81)

Average brick stress for each region is also given in parentheses.

## Discussion

The transition to an arctic environment and hypercarnivorous diet resulted in extremely rapid morphological evolution in the polar bear lineage. Our results indicate that the rate of cranial shape evolution in the polar bear lineage was at least twice as fast as in other branches of ursid phylogeny. Our estimate is probably conservative; while the phylogeny that we used for rate estimates dates the polar bear/brown bear split at ∼700 kya [3], recent analysis of sub-fossil polar bear remains suggests that polar bears diverged from brown bears as recently as 150 kya, and that the modern polar bear morphology was present by 130 kya [17]. Compared with other ursids, polar bears possess low flat skulls with elevated orbits [2], consistent with both semi-aquatic [18] and faunivorous [2] adaptations. This morphology might also increase hunting efficiency by allowing bears to thrust their heads into breathing holes or pupping dens. Polar bear evolution was facilitated by the expansion of polar ice sheets and floes in the late Pleistocene [19]. If polar bears evolved from coastal populations of brown bears [6], as molecular evidence now suggests [3]–[5], [17], then rapid evolution of adaptations for semi-aquatic life and hypercarnivory could have occurred to facilitate foraging over wider areas. Polar bears have denser fore- and hindlimb bones, a common adaptation of aquatic mammals, than closely related brown bears, further supporting this interpretation [20].

Although polar bears possess mechanically efficient skulls, as indicated by larger bite forces for a given muscle effort (Fig. 2A), we found that they also possess energetically inefficient and structurally weaker skulls (Fig. 2b; Fig. 3). This initially seems somewhat counterintuitive; among other carnivoran families, more carnivorous taxa tend to have stronger skulls [11], [13], [14]. However, polar bears feed almost exclusively on young ringed (*Pusa hispida*) and bearded (*Erignatus barbatus*) seals, which, at 68–250kg, are small prey in comparison to a ∼500 kg adult polar bear [21], [22]. As a result, cranial reinforcement may not be necessary as in hypercarnivores such as lions or wolves that regularly take prey larger than themselves [11], [13], [14]. The performance of the polar bear skull is particularly poor during bites with the post-canine dentition. (Fig 2b; Fig 3b–d; f–h). Polar bears exhibit reduced premolars and molars in comparison with most other ursids [1] but also lack the well-developed shearing blade-like teeth of hypercarnivores [1], [23]. In this respect they parallel insectivorous carnivorans, such as aardwolf (*Proteles cristata*), bat-eared fox (*Otocyon megalotis*) and sloth bear (*Ursus ursinus*) [1], [2]. Although convergence between a carnivore and insectivores also appears surprising, consideration of food material properties sheds light on this finding. Polar bears feed as almost exclusively on blubber and flesh that, unlike bone, require little or no processing prior to swallowing. If there is no selective advantage to maintaining large molars, they can be rapidly lost through the action of a few small mutations [24] or simple developmental mechanisms [25], [26]. Brown bears, in contrast, are generalized omnivores with unreduced dentitions [1], [2]. Although they consume animal protein when available, brown bears seasonally consume large amounts of plant material, including grasses, which require extensive mechanical breakdown and repeated skull loading prior to swallowing [27]. This is reflected in their larger molar grinding area, similar to other omnivorous ursids [1]. The lower peak stresses and higher work efficiency of the the brown bear cranium may result in part from the species' deep, vaulted and pneumatized forehead (see Fig. 3), a morphology that is characteristic of all herbivorous and omnivorous ursids [2]. Although pneumatized spaces are associated with reduced structural strength of the cranium [28], their presence is also associated with dissipation of regular, large peak masticatory loads in bone-cracking hyaenas and fossil canids [29]–[32]. The low, flat head of the polar bear, while advantageous for its semi-aquatic lifestyle and hunting behavior, reduces the ability of the cranium to withstand repeated large loads generated by bites made with the post-canine dentition.

The polar bear has become a flagship species for global climate change in recent years. Projected climate trends in coming decades will have profound effects on polar bear populations by decreasing the availability of suitable denning sites as well as eliminating much of the polar sea ice over which this specialized ursid forages for its seal prey [33]. Furthermore, climate-driven northward expansion of temperate ecosystems and their associated faunas [34] has already begun to facilitate the movement of brown bears into polar bear territory [35]. The continued survival of the polar bear in the face of global climate change will ultimately depend on a range of factors, including behavioral and physiological flexibility. Our findings, combined with those of earlier studies, shed some light on the potential dietary flexibility of polar bear. In response to a specialized diet of seal blubber, polar bears rapidly lost the large grinding molars and deep vaulted skull that characterize omnivorous ursids. This has not only resulted in a dentition that is less suited to diets requiring high levels of oral food processing but also, based on our results, a cranium that is less suited to bearing the associated loads. Small differences in cranial stress and strain are probably not alone sufficient to force a species to extinction. However, increased competition from northward advancing brown bear populations will also present a significant challenge. In areas where specialized arctic foxes (*Vulpes lagopus*) overlap with more generalized red foxes (*Vulpes vulpes*), red foxes actively displace arctic foxes and control prime feeding and denning areas [36], [37]. In this context, even the slight selective advantage provided by the superior mechanical performance of the brown bear's cranial shape, combined with a loss of molar grinding area, could be enough in such a setting to contribute to the exclusion of the polar bear. As a consequence of exceptional rapid specialization to a high arctic diet of seal flesh, the polar bear appears to have lost the generalized feeding abilities of its close relative. As a result, if current climate trends continue, one of the most striking examples of rapid phenotypic evolution may be lost as quickly as it appeared.

## Materials and Methods

### Rates of Cranial Shape Evolution

We computed multivariate rates of cranial shape evolution following [7]. Our morphometric data comprised mean principal component scores from a previous study of cranial shape variation across all extant ursid species [2]. We calculated rates of evolution from these data on an ursid phylogeny (Fig. 1) with topology and branch lengths from [3]. The rate of cranial shape evolution in the polar bear lineage was compared to the distribution of rates in other ursids using a one-tailed T-test. Analyses were conducted with R 2.10.1 [38] using custom-written scripts and functions from the APE [39] and Geiger [40] packages.

### Creating skull models

Dry skulls of one adult male polar bear (Illinois State Museum H001-05) and one adult male brown bear (United States National Museum 82003) were CT scanned at the High Resolution CT facility at the University of Texas, Austin. Slice thickness/inter-slice spacing was 0.75mm (polar bear) and 1mm (brown bear). Both scans are available via the digital morphology website (http://www.digimorph.org). Due to the high cost of CT scanning and the time consuming nature of FE model construction, only one specimen per species was used. Both specimens were quantitatively typical for their species based analysis of landmark data following [2]. Skulls were assessed as adult based on tooth eruption and full closure of the basilar synchondrosis.

3D surface models of the crania were rendered in AMIRA v.4.1.2-1 (Visualization Sciences Group, Massachusetts, USA). An automated thresholding tool was initially used to delimit bone surfaces. We then manually edited the slices. At this stage we made a number of simplifying steps to reduce model complexity. First, we omitted the complex turbinal bones within the nasal cavity and semi-circular canals of the middle ear, as these presumably do not function in load bearing. We also simplified the morphology of complex structures that can be problematic in FE modeling, such as the perforated cribriform plate of the ethmoid. Second, we modeled teeth as continuous with surrounding maxillary bone as in other FE studies [9]–[11], [13], [14], [41]. Although tooth roots and periodontal ligaments (PDL) play important roles in transmitting and absorbing forces, recent work suggests that inclusion of the PDL in finite element models affects only local strain in the region of the alveolus [42] and so presumably does not affect global patterns of performance. Third, we ignored the distribution of trabecular bone and modeled the entire cranium as continuous cortical bone. Although this will over-stiffen the models, both contained similar amounts of cancellous bone and so are presumably affected in similar ways. Finally, we omitted to model the intricate three-dimensional morphology of cranial sutures. Recent FE work suggests that sutures may play important roles in locally reducing strain in non-mammalian tetrapod skulls [43], [44] but their significance in mammalian cranial function remains to be explored. Internal cavities, such as the frontal sinuses and tympanic bulla cavity were modeled as hollow, preserving potential biomechanical function. Simplified skull models were imported into Geomagic Studio v.10. (Geomagic Corp. North Carolina, USA), where we manually edited the surfaces to correct artifacts of the reconstruction process and patch holes. Once watertight surface models of the two skulls had been created, we re-exported them for FE modeling.

### Finite Element models

The complete, simplified skull models were imported into Strand7 (Strand7 Pty. Ltd., Sydney, Australia) for FE analysis. We created Finite Element meshes of the cranium only (the mandible was retained only for positioning muscle vectors) using 4-noded tetrahedral elements. The final models comprised 841,531 elements for the polar bear and 984,184 elements for the brown bear. Complete finite element models have been deposited with, and are available for download from Biomesh (http://www.biomesh.org/models). Ideally, we would have assigned complex material properties to our models to account for regional variation in the distribution of cortical and cancellous bone, and the orthotropic material behavior of bone. Because the use of homogenous material properties in FEMs has been shown to produce surface strains that fall within the range of values of obtained from *in vivo* strain gauge studies [45] and material properties are currently not available for polar or brown bear cranial bone, we made the simplifying assumption here to assign homogeneous isotropic material properties based on values for domestic dog cortical bone, following [46], [47] (E = 13.7 GPA, ν = 0.3). Our study is not validated and thus absolute values of results should be treated with caution. However, as the aim of our study is comparative and both skulls were modeled in identical ways, we should still be able to draw broad conclusions regarding comparative performance of the skulls of the two species from the results obtained.

### Muscle forces

We applied muscle forces over the origins of the temporalis, masseter and pterygoideus (internal+external) muscles using the tangential-plus-normal traction model in the program BoneLoad [15]. This method incorporates the effects of muscle wrapping around curved bone surfaces and eliminates artifacts caused by point loads in areas of muscle insertion. A thin layer of plates (10^−4^mm) was applied over the entire area of muscle origin for each muscle. The plates were assigned the same material properties as the tetrahedral elements forming the cranium. Muscle forces were then applied to the plate surfaces. To provide focal points for the muscle forces to act towards, we identified the *x,y,z* coordinates of nodes on the mandibles representing the estimated center points of the temporalis, masseter, and pterygoideus insertion areas. We subsequently deleted the plates constituting the mandibles and, in their place, created six nodes at the exact co-ordinates of the previously identified focal positions for the muscles. These newly created nodes were used as focal points for the action of the muscle forces. For example, all left temporalis forces pulled towards a focal node representing the center point of temporalis insertion on the left mandible.

Measurements of cross section area of the jaw muscles were not available for the species modeled here. Instead, a total amount of muscle force (see below) was distributed in each model according to percentage contribution of temporalis (65.17%), masseter (28.08%) and pterygoideus (6.75%) to total jaw muscle mass in the closely related American black bear *Ursus americanus* [48]. Available evidence suggests that these values are consistent across such disparate carnivoran families as felids and canids [48], [49]. During biting with the post-canine dentition, jaw muscles are likely to differ in activity patterns on working and balancing sides of the jaw. To ensure that the assumption of maximal muscle activity did not bias our results, we conducted analyses at the post-canine dentition with forces allocated at a 1∶0.66 ratio between working and balancing sides, based on electromyographic work on the domestic dog [50] and subsequent FE studies of carnivoran mastication [31].

### Constraints

To prevent free-body rotation (unconstrained movement of models in space), we followed protocols described in [45]. We constrained a single node at each glenoid fossa, creating a virtual axis of rotation with the ventrally directed muscle forces rotating the cranium about the temporal-mandibular joint. To simulate biting behavior and measure feeding performance (i.e. bite force, cranial stress and strain), we applied additional single node constraints at teeth involved in the simulated biting behaviors. This added a virtual bite point, with the rotating skull meeting a point of resistance at the bite point and a resultant virtual bite force generated. From this action, resultant stresses and strain can be calculated and visualized. We simulated four bite scenarios that ursids use when feeding: a bilateral canine bite (one constrained node at the tip of each canine); and unilateral bites at the fourth upper premolar - the “carnassial” (a single constrained node at the carnassial notch); upper first molar (a single constrained node at the protocone); and upper second molar (a single constrained node at the protocone). To ensure that asymmetries in the models and placement of constraints did not influence results, we repeated analyses for both left and right teeth and averaged subsequent bite force and strain energy results (Table 2).

### Scaling and Assessing Performance

We controlled for differences in size between the models using a recently developed method for comparing FE models [16]. In order to ensure that stress values are comparable among models of different sizes, it is important that force to surface area ratios are constant among finite element models. Therefore, prior to analysis, both models were scaled to common surface area corresponding to that of the polar bear model (1,209,042 mm^2^). To produce realistic estimates of bite force we used the total muscle force derived from a dry skull estimate of the cross sectional area of temporalis and masseter plus pterygoideus muscles in the polar bear (18069.6 N - [51]). For bilateral canine bites, this total muscle force was distributed according to proportions described above from the black bear. For unilateral post-canine bites, we reduced the balancing side muscle forces by 2/3, resulting in a working side total of 9034.8 N and a balancing side total of 6023.2 N.

We evaluated performance of the models based on three criteria. First, we determined how skull shape affects bite performance by comparing bite forces at the constrained nodes on the teeth. Because all models were scaled to a common surface area and used equal muscle forces, our null hypothesis was that bite forces should be identical among the models. Any differences in bite forces could then be interpreted as the result of differences in skull geometry alone [16]. Second, we assessed strength of the skull models by comparing model stress, measured as Von Mises stress [9]. Bone is an elastic material and therefore fails under a ductile, rather than brittle model of fracture [52]. Von Mises stress is a scalar function of the principle stresses at each element and provides a good predictor of failure due to ductile fracture [9]. Lower peak stress values and more even stress distributions were interpreted as indicating a stronger structure for a given loading condition. Finally, we assessed the work efficiency of the skull models by comparing total strain energy values, a measure of energy lost to deformation. In terms of work efficiency, efficient structures are those that maximize stiffness for a given volume of material [16]. Lower strain energy values indicate stiffer structures and therefore greater work efficiency. Strain energy values were corrected for differences in volumes of the models using Equation 5 from Dumont et al. [16]. Our null hypotheses for all analyses were that stress and strain energy values should be identical among scaled models. All FE analyses were linear static and were completed in Strand7.
